# Colles’s Fracture1Read before the Buffalo Academy of Medicine, December 6, 1898.

**Published:** 1899-03

**Authors:** E. M. Dooley

**Affiliations:** Buffalo, N. Y., Attending surgeon Buffalo Hospital Sisters of Charity and Emergency Hospital; surgeon to the Erie Railroad Company


### COLLES'S FRACTURE.[1]

E. M. DOOLEY, M. D., Buffalo, N. Y.,
Attending surgeon Buffalo Hospital Sisters of Charity and Emergency Hospital; surgeon to the Erie Railroad Company.

THE comparative frecuency of fractures, according to compiled statistics, is misleading. It is impossible to obtain reports, excepting from hospitals or dispensaries, where only a fractional part of all cases apply for treatment. All will agree with me, however, when I state that Colles's fracture is the most frequent met with in general practice.

The simple manner of its occurrence may easily account for this frequency. The patient, walking on an icy sidewalk, suddenly loses his footing and falls heavily to the ground; instinctively he throws out his hand, in prone position, with palms spread, and extending backward, receiving the weight of his body upon the wrist in its most fixed position. The upper surfaces of scaphoid and semilunar are convex and closely fitted to the concave surface of the lower end of the radius. The firm support given by the carpal bones accounts for the fracture, which usually occurs within an inch or two of the articulating surface. That the fracture should take place at this particular point, it has been observed, "is probably due to the character of the internal structure of the bone in this situation. The expanded lower end of the radius is mostly cancellous, while the shaft which joins it possesses a greater amount of compact tissue."

1. Read before the Buffalo Academy of Medicine, December 6, 1898.

The lower end of the ulna, although attached to the radius by short and comparatively strong ligaments, has a space existing between its articulatin surface and the cuneiform bone, occupied by the triangular cartilage and its synovial sacs. The strong lateral ligament is attached to a depression in the styloid process. When the radius gives way the strain comes upon the lower end of the ulna, which, having no direct support from the carpal bones, becomes dislocated downward. The lateral ligament tears the tip off the styloid process, leaving a roughened and sharpened extremity, which lacerates the ligaments and surrounding tissues. If this were all that happened in Colles's fracture, the treatment would be simple and result more satisfactory.

An eminent anatomist has wisely observed: "It is of equal importance to remember that in all fractures, in whatever region they occur, the bones are not the only tissues which suffer from the injury. There is always accompanying the breach in the continuity of the bones more or less damage to the soft structures about them. The interosseus ligament and periosteum with their special vessels must be lacerated and although in one sense this is essential for the process of repair, yet it presents a factor which probably accounts for the frequent unsatisfactory results."

The structures frequently involved are the tendons and nerves. We have about the wrist twenty-four tendons, whose functions not only deal with the movements of the wrist, but especially with movements of fingers and thumbs. Since the tendons dealing with the movements of the fingers are arranged into groups and surrounded by lubricating sheaths, it is of special moment whether they suffer from any undue violence at or subsequent to the injury to the bones, and since movement of these tendons are not apt to displace the fragments, there is no reason why passive motion of them should be delayed.

The nerves about the wrist consist of comparatively large trunks or branches. One in particular has a ganglionic enlargement, the termination of the posterior interosseus, which lays beneath the muscles and rests in close proximity to the interosseus membrane and posterior surface of the wrist joint. The ulna nerve passes over the anterior surface of the bone and to the outer side of the joint where the head of the ulna presses forward, when it becomes dislocated. The median nerve passes between flexor sublimus and flexor profundus, while the anterior interosseus and radial nerves terminate more superficially. Besides these larger trunks cutaneous branches supply the integument and they too are in evidence when neuritis happens to be one of the complications attending the treatment of Colles's fracture. The arrangement of the superficial veins, especially those on the dorsal part of the hand and wrist, marks another structure which must be taken into consideration in the treatment of injuries to the wrist. "They play a valuable part at nature's efforts at repair and should not be interfered with."

I have mentioned these anatomical points because I consider that the complications apt to arise in the course of the teatment of Colles's fracture are due not so much to the direct effect of the fracture itself as to the injury to the surrounding structures. At the time the fracture occurs these tissues may not be directly injured, and subsequently give much trouble, due possibly to the means taken to produce immobility of the fragments, that perfect union may ensue. Hence in our treatment we should remember the surrounding structures, if we would have ideal results.

In seeking to repair a Colles's fracture our efforts should be directed: (1) Toward a complete reduction of both fractured radius and dislocated ulna. So essential is this that if successfully accomplished the treatment is usually uncomplicated and the result all that one could desire. (2) The use of simple, but effective means to keep the fragments in apposition and to prevent a recurrence of the dislocation of the ulna, being careful always not to have any undue pressure to interfere with the return circulation. (3) Prevent adhesions of tendons by early passive motion of fingers, thumb and wrist. (4) Removal of the mechanical appliance as soon as practicable after the fragment has united.

In the treatment, then, the first question is, how is complete reduction of the fracture and dislocation best accomplished? For this purpose one assistant at least is necessary. With the assistant holding the upper part of the forearm firmly, the patient's elbow flexed and the hand in prone position, the surgeon stands facing the end of the patient's hand, he can then grasp the patient's wrist and make traction and forcible flexion, using his fingers to manipulate the fragments of the radius and replace the lower end of the ulna. If two assistants are at hand, better still, one can make traction and forcible flexion while the other holds the forearm, the surgeon being free to manipulate the fragments. If we fail to reduce the fracture properly, then I think an anesthetic should be administered. Reduction of the fracture and dislocation is the important step, and every means should be employed even to the use of an anesthetic to accomplish this purpose.

The next step in the treatment is the use of a simple appliance to produce immobility. For this purpose I have found none more convenient than the anterior metallic splint, shaped to fit the hand in the flexed position. The third step in the treatment is to prevent adhesions of the tendons and ankylosis of the wrist, by early and frequent passive motion. Formerly it was thought necessary to retain the hand and wrist in a fixed position for several weeks before an attempt should be made to produce passive motion. Fortunately, opinion in this respect has undergone a radical change and at the present time it is more wisely taught that passive motion should begin not later than one week after the injury, and continue at regular intervals until the patient has fully recovered. I think Moullin's advice about passive motion is the safest to follow. He advises movements of the fingers from the first day, careful movement of the wrist from the fourth or fifth day. He also says: "But not uncommonly it is necessary to place the patient under an anesthetic and work every joint thoroughly before there is much improvement."

When shall we remove the splint? My experience has taught me it is best to remove the splint in most cases about the end of the third week. In this, however, the surgeon must be guided by the result. Occasionally he will find it necessary to retain the splint a week or so longer, and at times at the exhibition of unusual stiffness he may deem it more advisable to dispense with its use after two weeks. No set rule will fit every case as regards this matter, and we must be guided by conditions as we find them, keeping in mind always that our main object in the treatment of Colles's fracture is to obtain not only union without deformity, but also without stiffness.

Unfortunately Colles's fracture has its complications, and no matter how intelligently we may treat them we are bound to have some apparently bad results. Contributing to this not a little is the age and condition of the patient. For our own protection it is always wise to learn the true condition of the patient whenever called to treat injury to the bones. Especially is it well to note whether he suffers from any specific disease, or has he a tubercular history, or rheumatic or gouty diathesis. A true history as to how the accident occurred is also essential. Did he receive the injury by simply falling on a walk or from a considerable height, as from a building, or was it possibly due to direct violence by having a heavy weight fall upon the wrist. All this is necessary to determine the severity of the injury and enable us not only to treat it with a corresponding care, but also to give a more accurate prognosis. Occasionally the patient suffers from a double Colles's fracture and it is a good practice to always examine both wrists. The ulna may have a compound dislocation. The question is how shall we treat it? Possibly if the head of the ulna is much injured it may be necessary to remove it. But with the cases I have seen, good results have followed by carefully rendering the lacerated tissues aseptic and returning the bone to its former position and closing with sutures. When the fracture is compound, it is usually due to direct violence and it is a difficult matter to render it aseptic on that account; still I think an effort should always be made to treat it in this manner. Until recently I have not had a case complicated with neuritis. But at the present time I have one such under observation. In the acute stage the patient suffered the most intense pain, the tissues became edematous, and passive motion was out of the question. With the administration of opiates I was partly enabled to subdue the pain, but it required unusually large doses, repeated every two hours, to enable the patient to obtain part of a night's rest. When the pain and swelling had somewhat subsided, I gave the patient chloroform and carefully used passive motion and was enabled in this way to break up adhesions, but as the patient objects to taking an anesthetic again, I watch him closely, to see that he moves his fingers and wrist, with the prospect of obtaining a fair result.

One distinctive feature about Colles's fracture is its location. It is before the patient's eyes, and where his friends and relatives can observe and note any existing deformity. It is for our especial interest therefore to give it every possible attention and never to treat it lightly, that our results may be a credit, not only to ourselves, but to our profession.

406 LOUISIANA STREET.

---

THE TREATMENT OF DIARRHEA BY DERMATOL.—Dr. Clemthal, of Helsingfors, has experimented upon the action of dermatol in sixty cases of diarrhea (related in the *Therapeutic Gazette*) dependent upon different causes, and in all of them obtained equally good results with those obtained by the use of opium and subnitrate of bismuth. The doses which he gave varied from four to seven grains, four to six times in twenty-four hours. In no case did he observe that the prolonged use of dermatol produced any inconvenient result.—*Maryland Medical Journal.*

